# Pesticide Residues in Honey from the Major Honey Producing Forest Belts in Ghana

**DOI:** 10.1155/2017/7957431

**Published:** 2017-08-16

**Authors:** Godfred Darko, Jonah Addai Tabi, Michael Kodwo Adjaloo, Lawrence Sheringham Borquaye

**Affiliations:** ^1^Department of Chemistry, Kwame Nkrumah University of Science and Technology, Kumasi, Ghana; ^2^Technology Consultancy Centre, Kwame Nkrumah University of Science and Technology, Kumasi, Ghana

## Abstract

Concentrations of pesticides residues in honey sampled from the major honey producing forest belts in Ghana were determined. Samples were purposively collected and extracted using the QuEChERS (Quick, Easy, Cheap, Effective, Rugged, and Safe) method and analysed for synthetic pyrethroids, organochlorine, and organophosphate pesticide residues. Aldrin, *γ*-HCH, *β*-HCH, ∑endosulfan, cyfluthrin, cypermethrin, deltamethrin, permethrin methoxychlor, ∑DDT, chlorpyrifos, fenvalerate, malathion, dimethoate, and diazinon were all detected at the concentration of 0.01 mg/kg, while cyfluthrin and permethrin were detected at mean concentrations of 0.02 and 0.04 mg/kg, respectively. All the pesticide residues detected were very low and below their respective maximum residue limits set by the European Union. Hence, pesticide residues in honey samples analyzed do not pose any health risk to consumers.

## 1. Background

Honey, the sweet and viscid fluid, which is produced by honeybees from the nectar of flowers, contains significant amounts of mineral matter, vitamins, and enzymes [1]. With respect to carbohydrates, honey is mainly fructose (about 38.5%) and glucose (about 31.0%) [2] and is known to be a healthier nutritional choice than sugar [3, 4]. However, the specific composition of any batch of honey and contaminants present is dependent on the crops from which the nectar was sourced and the surroundings of the beehive. Honey is widely used for both nutritional and medicinal purposes and it is known to have therapeutic actions against infections, wounds, and cancers [5]. It has been used to treat cough and sore throat, ulcer, earache, measles, and eye diseases [6]. It is consumed, worldwide, as food or medicine. It is known that feeding infants with honey helps to improve their memory and growth, reduce anxiety, and enhance the children's performance as they grow in life [7]. Honey is also used in cosmetics and as a natural sweetener in food manufacturing.

While the nutritional value and quality aspects of honey are important, assurance of its chemical safety is critical to consumer acceptance. The health and nutritional benefits of honey are reduced if they are contaminated with toxic chemicals such as residues of pesticides [8] and other environmental contaminants. Honey is prone to contamination from the environment as trace amounts of pollutants, including residues of pesticides, brought into the hive by the honeybee get concentrated during processing of the honey.

Pesticides are extensively used in Ghana [9], mainly for agriculture and disease control. While the use of the synthetic pyrethroids and carbamates as insecticides, weedicides, and fungicides is on the increase, the use of the organochlorines (mostly as insecticides on cash crops) has drastically reduced since their ban in 1999 [10]. However, residues of organochlorine pesticides continue to be detected in environmental samples and food items [11, 12] due to their persistence in the environment and illegal use. Even though the newer generation pesticides (synthetic pyrethroids, organophosphorus, and carbamates) are not as persistent as the first generation organochlorines, they are much more acutely toxic. Residues of organophosphorus pesticides have been detected in fruits and vegetables sold on the Ghanaian market [9].

All pesticides are toxic and several of them are potential carcinogens which may cause chromosomal abrasions [13]. Pesticides are also known to cause changes in the endocrine [14], the reproductive [15–17], and the nervous systems [18, 19]. Beekeeping is being promoted nation-wide, as a source of additional income for farmers. Therefore, monitoring of pesticide residues in the commodity is essential to ensure its quality and safety. However, there is no published data on the extent of pesticide contamination in honey in the country. Hence, this work determined the concentrations of pesticides residues in honey in the major honey producing forest belts in Ashanti, Brong-Ahafo, and Western regions of Ghana. Therefore, this study gives the baseline and the first ever reported data on the levels of pesticide residues in honey from Ghana.

## 2. Materials and Methods

### 2.1. Sampling

**Figure 1:**
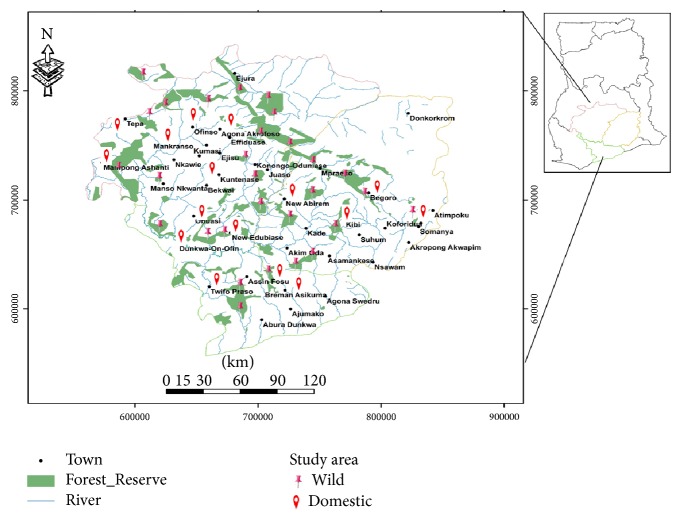
Map of Ghana showing the sampled zones in the Ashanti, Brong-Ahafo, and Western Regions.

Honey samples were purposively collected from the major honey producing forest belts in Ashanti, Brong-Ahafo, and Western Regions of Ghana (Figure 1). All the sites are located in agricultural farmlands where various pesticides are applied continuously to either control insects or weeds. Honey from both wild forest and beekeepers was collected from February to June 2014 within the harvesting season. Protective gowns were worn and smoke from fire was used to drive away the honeybees prior to sample harvesting. After harvesting, the honey was squeezed from the wax, followed by filtration to remove debris. In all, a total of 45 honey samples consisting of 30 from the wild forest and 15 from domesticated beehives were obtained. About 500 mL of each sample was placed in labeled plastic containers and sent to the laboratory for analysis. All samples were kept at ambient temperature until the analysis.

### 2.2. Extraction

All reagents used were of analytical grade and were used as obtained without further purification. Samples were extracted using the Quick, Easy, Cheap, Effective, Rugged, Safe (QuEChERS) multiresidue method for the analysis of pesticide residues in low fat matrix [20–22]. A 5 g portion of homogenized honey sample was spiked with 100 *µ*L internal standard, mixed with 10 mL of ultrapure water (resistivity 18.2 MΏ) and homogenized by shaking to reduce its viscosity and facilitate its handling. The sample was mixed with 10 mL of acetonitrile (Sigma Aldrich, St. Louis, MO, USA) and subjected to extraction by shaking for 3 min. A mixture of salts composed of 1 g sodium chloride, 1 g disodium hydrogen citrate sesquihydrate, 0.5 g trisodium citrate dehydrate, and 4 g magnesium sulphate anhydrous (all from M&B Chemicals, New Delhi, India) were added to the mixture and vortexed for 3 mins for extraction with separation.

The organic phase was separated from the inorganic phase after centrifugation at 3000 rpm for 5 mins. The supernatant was collected and the residue was reextracted with 10 mL of the solvent. The extract was transferred into a single-use polypropylene centrifuge tube, which contains 25 mg primary-secondary amine (PSA) and 150 mg MgSO_4_ (Sigma Aldrich, St. Louis, MO, USA). The tube was vortexed for 1 min followed by centrifugation at 3000 rpm for 5 mins. After the centrifugation, the cleaned extract was transferred into a screw cap vial and the pH adjusted to 5 using 5% (v/v) formic acid solution in acetonitrile [23–27]. The pH-adjusted extracts were filled into vials for gas chromatography. The innovation in this method lies in the fact that it combines the extraction and clean-up processes into one-step, thereby reducing cross-contamination and increasing throughput [28].

### 2.3. Gas Chromatography

Analysis was carried out on a Varian CP-3800 gas chromatograph (Brescia, Italy) with a CombiPAL autosampler, an electron capture detector (ECD) for organochlorine and synthetic pyrethroids pesticides as well as pulse flame photometric detector (PFPD) for organophosphorus pesticides.

### 2.4. Chromatographic Conditions for Organochlorine and Synthetic Pyrethroids

Separation of analytes was achieved on a Varian capillary column (30 m + 10 m EZ Guard × 0.25 mm internal diameter fused silica capillary coated with VF-5ms, 0.25 *μ*m film). The carrier gas was nitrogen (99.999% purity) at a flow rate of 1 mL/min. Oven temperature was maintained initially at 70°C for 2 min and increased at 25°C/min to 180°C then at 5°C/min to 300°C. The injection volume was 1 *µ*L, injected in splitless mode at an injection temperature of 270°C, whilst the ECD detector was maintained at 300°C.

### 2.5. Chromatographic Conditions of Organophosphorus Pesticides

Organophosphorus pesticides were separated on Varian capillary column (30 m × 0.25 mm internal diameter fused silica capillary coated with VF-1701ms, 0.25 *μ*m film) but detected and quantified on a PFPD kept at 280°C. The carrier gas was nitrogen (99.999% purity) at a flow rate of 2 mL/min. Oven temperature was maintained initially at 70°C for 2 min and increased at 25°C/min to 200°C/min and then at 20°C/min to 250°C. The injection volume was 1 *µ*L, injected in splitless mode at an injection temperature of 270°C.

### 2.6. Quality Control

Recovery test was carried out by spiking blank samples with 0.02 mg/kg standard mix. The fortified samples were then allowed to equilibrate for 30 min prior to extraction and analysis using the analytical methods described. The mean recovery values were calculated from the peak area obtained. Identification of analytes was by comparison with the retention times of the standards. Retention times were within ±0.20 min of the expected retention times [28]. Quantification of analyte concentrations were based on 5-point (ranging from 0.01 to 2.00 mg/kg) calibration curves prepared. Blanks were routine run to check and correct instrument drifts and column contamination. All analyses were done in triplicate.

### 2.7. Limits of Detection

The detection limits of the GC coupled with either ECD or PFPD were determined for each pesticide by injecting serially diluted standard mix. Detection limits of the method were found by determining the lowest concentrations of the residues in each of the matrices that could be reproducibly measured at the operating conditions of the GC using a signal-to-noise ratio of three. The detection and quantification limits for all the pesticide categories were found to be 0.01 and 0.04 mg/kg, respectively. Blank analyses were also performed in order to check interference from the sample. All analyses were done in triplicate.

## 3. Results and Discussion

 Recovery of the pesticides from the spiked samples was 90–99% for the organochlorines, 94–98% for the organophosphorus, and 89–96% for the synthetic pyrethroids. Recovery range of about 75–105% is deemed satisfactory in pesticide residue analysis [26, 27]. Therefore, the recoveries achieved in this study were satisfactory indicating the extraction method used was selective and the analytical/instrument conditions were sensitive to the analytes.

In all, eleven pesticides (diazinon, chlorpyrifos, dimethoate, methoxychlor, malathion, aldrin, cyfluthrin, permethrin, fenvalerate, endosulfan, and DDT) were detected in the honey samples (Table 1). The concentrations found were mostly close to the limits of detection. Chlorpyrifos, dimethoate, methoxychlor, and malathion were the only organophosphorus detected but none of them had a concentration higher than the method's detection limits or the EU MRL. Cyfluthrin and permethrin were the most detected pyrethroids.

Samples from the Ashanti region had traces of dimethoate (0.01 mg/kg), endosulfan (0.01 mg/kg), fenvalerate (0.01 mg/kg), and DDT (0.01 mg/kg). Concentration of ∑DDT was equal to the maximum residue limit (MRL) and thus warrants attention. The concentrations of fenvalerate and dimethoate were close to their MRL of 0.02 mg/kg. Concentration of total endosulfan was, however, far less than the MRL of 0.05 mg/kg. The forest honey samples showed a similar concentration pattern. Concentrations of cyfluthrin and methoxychlor were the same as their MRL and those of fenvalerate and permethrin were close to their MRL, whiles that of chlorpyrifos was lower than its MRL.

In the Brong-Ahafo region, diazinon, malathion, and permethrin were detected at concentrations higher than the method detection limits. However, the concentrations of malathion and permethrin (0.01 mg/kg) were lower than their MRL of 0.02 and 0.05 mg/kg, respectively. The concentration of diazinon detected was the same as its MRL of 0.01 mg/kg. In the forest samples, methoxychlor, aldrin, and cyfluthrin had the same concentrations as their MRL. Concentrations of the other residues detected (chlorpyrifos, permethrin, and endosulfan) were less than their MRL.

A similar case can be made for samples from the Western region. The concentrations diazinon and DDT detected were the same as their MRL (0.01 mg/kg). The concentration of malathion (0.01 mg/kg) was half its MRL. In the forest samples, aldrin (0.01 mg/kg) and cyfluthrin (0.02 mg/kg) had concentrations that were the same as their MRL. Whereas the concentration of permethrin (0.04 mg/kg) was close to its MRL (0.05 mg/kg), that of fenvalerate (0.01 mg/kg) was half its MRL. The forest samples recorded more pesticides than the beehive samples in all the regions. Of the pesticides detected, methoxychlor, chlorpyrifos, malathion, dimethoate, and diazinon belong to the organophosphorus group which is the most commonly used in the country.

Concentrations of organochlorine residues obtained in the current work are lower than the 0.09 mg/kg HCB and 0.143 mg/kg ∑DDT reported in honey samples from central Portugal [8]. They were also lower than the 0.30 *µ*g/g HCB, 0.05 *µ*g/g chlordane, 0.04 *µ*g/g heptachlor, 0.06 *µ*g/g aldrin, and 0.36 *µ*g/g total endosulfan reported for honey samples from Kahramanmaras, Turkey [29].

The ban on the use of DDT and restrictive use of other organochlorine pesticides such as endosulfan seem to have had a positive effect on environmental contamination even though traces of pesticide residues used in the past are still being detected in the environment. The presence of pesticide residues in the environment is a major cause of honeybee loss [30, 31].

According to European Union (EU) regulations, honey as a natural product must be free of any chemical contaminants and safe for human consumption [8]. On this basis, all the samples analyzed agreed with this regulation. However, since consumers are exposed to pesticides, usually in minute quantities, through several different food groups such as fruits, honey, and vegetables, monitoring the various food items becomes imperative in assessing environmental and human health risks. The significance of the study is that the practitioners of the honey industry could be made aware of the possibility of pesticide contamination in their honey products and thus take steps to avert it. Perhaps the relevant authorities could be involved in the regulation of pesticides use in the country.

## 4. Conclusions

QuEChERS method was satisfactorily applied for the extraction of pesticide residues in honey from major honey producing forest belts in Ghana. Aldrin, *γ*-HCH, *β*-HCH, ∑endosulfan, cyfluthrin, cypermethrin, deltamethrin, permethrin methoxychlor, ∑DDT, chlorpyrifos, fenvalerate, malathion, dimethoate, and diazinon were all detected but at very low concentrations. All the pesticide residues had concentrations lower than the recommended EU maximum residue limits. Hence, honey samples analyzed do not pose any health risk to the consumer as far as pesticide residues are concerned.

## Figures and Tables

**Table 1:** Concentrations of pesticides.

		Average concentrations (mg/kg) of different classes of pesticide residues in the 3 different regions						MRL (EU, 2005)
		Ashanti		Brong-Ahafo		Western		
		Beehive	Forest	Beehive	Forest	Beehive	Forest	
Organophosphorus	Chlorpyrifos	<0.01	0.01	<0.01	0.01	<0.01	<0.01	0.05
	Diazinon	<0.01	<0.01	0.01	<0.01	0.01	<0.01	0.01
	Dimethoate	0.01	<0.01	<0.01	<0.01	<0.01	<0.01	0.02
	Fenitothion	<0.01	<0.01	<0.01	<0.01	<0.01	<0.01	0.01
	Malathion	<0.01	<0.01	0.01	<0.01	0.01	<0.01	0.02
	Methamidophos	<0.01	<0.01	<0.01	<0.01	<0.01	<0.01	0.05
	Parathion	<0.01	<0.01	<0.01	<0.01	<0.01	<0.01	0.01
	Phorate	<0.01	<0.01	<0.01	<0.01	<0.01	<0.01	0.05
	Phosmet	<0.01	<0.01	<0.01	<0.01	<0.01	<0.01	0.05
	Pirimiphos-methyl	<0.01	<0.01	<0.01	<0.01	<0.01	<0.01	0.05
	Profenofos	<0.01	<0.01	<0.01	<0.01	<0.01	<0.01	0.05

Synthetic pyrethroids	Bifenthrin	<0.01	<0.01	<0.01	<0.01	<0.01	<0.01	0.05
	Cyfluthrin	<0.01	0.02	<0.01	0.02	<0.01	0.02	0.02
	Cypermethrin	<0.01	<0.01	<0.01	<0.01	<0.01	<0.01	0.05
	Deltamethrin	<0.01	<0.01	<0.01	<0.01	<0.01	<0.01	0.05
	Fenvalerate	0.01	0.01	<0.01	<0.01	<0.01	0.01	0.02
	Permethrin (cis/trans)	<0.01	0.04	0.01	0.04	<0.01	0.04	0.05
	*λ*-Cyahalothin	<0.01	<0.01	<0.01	<0.01	<0.01	<0.01	0.02
	∑Permethrin	<0.01	0.04	0.01	0.04	<0.01	0.04	0.05

Organochlorines	Aldrin	<0.01	<0.01	<0.01	0.01	<0.01	0.01	0.01
	Dieldrin	<0.01	<0.01	<0.01	<0.01	<0.01	<0.01	0.01
	*γ*-Chlordane	<0.01	<0.01	<0.01	<0.01	<0.01	<0.01	0.01
	Heptachlor	<0.01	<0.01	<0.01	<0.01	<0.01	<0.01	0.01
	Lindane	<0.01	<0.01	<0.01	<0.01	<0.01	<0.01	0.01
	Methoxychlor	<0.01	0.01	<0.01	0.01	<0.01	<0.01	0.01
	*α*-HCH	<0.01	<0.01	<0.01	<0.01	<0.01	<0.01	0.01
	*β*-HCH	<0.01	<0.01	<0.01	<0.01	<0.01	<0.01	0.01
	∑DDT	0.01	<0.01	<0.01	<0.01	0.01	<0.01	0.01
	∑Endosulfan	0.01	<0.01	<0.01	0.01	<0.01	0.01	0.05

∑Endosulfan = sum of isomers; ∑Permethrin = sum of cis- and trans-isomers; ∑DDT = sum of *p,p*′ DDT, *o,p*′*-*DDT, and *p,p*′*-*DDE.

## Abbreviations

MRL: Maximum residue limits
EU: European Union
QuEChERS: Quick, Easy, Cheap, Effective, Rugged, and Safe
PSA: Primary-secondary amine
PFPD: Pulse flame photometric detector
HCH: Hexachlorocyclohexanes
GC-ECD: Gas chromatograph-electron capture detector
∑Permethrin: Sum of cis- and trans-isomers of permethrin
∑DDT: 1,1,1-Trichloro-2,2-di-(4-chlorophenyl)ethane.
